# Quantification of total haemoglobin concentrations in human whole blood by spectroscopic visible-light optical coherence tomography

**DOI:** 10.1038/s41598-019-51721-9

**Published:** 2019-10-22

**Authors:** Colin Veenstra, Saskia Kruitwagen, Dafne Groener, Wilma Petersen, Wiendelt Steenbergen, Nienke Bosschaart

**Affiliations:** Biomedical Photonic Imaging Group, Faculty of Science and Technology, Technical Medical Centre, University of Twente, P.O. Box 217, 7500 AE Enschede, The Netherlands

**Keywords:** Applied optics, Biophysics

## Abstract

The non-invasive quantification of total haemoglobin concentrations [tHb] is highly desired for the assessment of haematologic disorders in vulnerable patient groups, but invasive blood sampling is still the gold standard in current clinical practice. This work demonstrates the potential of visible-light spectroscopic optical coherence tomography (sOCT) for quantifying the [tHb] in human whole blood. To accurately quantify the [tHb] from the substantial optical attenuation by blood in the visible wavelength range, we used a combination of zero-delay acquisition and focus tracking that ensures optimal system sensitivity at any depth inside the sample. Subsequently, we developed an analysis model to adequately correct for the high scattering contribution by red blood cells to the sOCT signal. We validate our method and compare it to conventional sOCT (without focus tracking and zero-delay acquisition) through *ex-vivo* measurements on flowing human whole blood, with [tHb] values in the clinical range of 7–23 g/dL. For our method with optimized sensitivity, the measured and expected values correlate well (Pearson correlation coefficient = 0.89, p < 0.01), with a precision of 3.8 g/dL. This is a considerable improvement compared to conventional sOCT (Pearson correlation coefficient = 0.59, p = 0.16; precision of 9.1 g/dL).

## Introduction

Haemoglobin is the oxygen transporting protein in red blood cells (RBCs) and exists mainly in two forms: oxyhaemoglobin (HbO_2_) and deoxyhaemoglobin (Hb), which together account for the total amount of haemoglobin (tHb). Current clinical methods that measure total haemoglobin concentrations [tHb] in blood require invasive blood sampling, which are frequently used for diagnosing haematologic disorders such as anaemia and polycythaemia^1^. While invasive blood sampling is fast and highly accurate, it cannot be used for continuous monitoring for acute and intensive care purposes^2^. In premature infants, invasive blood sampling is associated with increased risk of infection and significant blood loss. For this vulnerable patient group, frequent blood sampling may in fact be the cause, rather than the prevention of anaemia^3^. Hence, there is an obvious need for the non-invasive assessment of [tHb].

Currently, non-invasive optical techniques are being developed for measuring [tHb], which make use of the high optical absorption of haemoglobin in the visible and near-infrared wavelength range. These techniques include photoplethysmography and photoacoustics. However, the precision of photoplethysmography is limited by the uncertainty in the exact optical path length of the detected photons^4^, and the precision of photoacoustic [tHb] determinations remains to be determined^5^. Therefore, we follow an alternative approach by using visible-light spectroscopic optical coherence tomography (sOCT) for non-invasive quantification of [tHb]. The unique advantage of sOCT is that the optical path length (OPL) inside tissue is both known and controllable. This allows for both quantitative and spatially confined measurements of chromophore concentrations *in-vivo*^6^, and potentially enables [tHb] determinations within individual blood vessels - without any cross-talk from the optical attenuation by surrounding tissue structures.

Visible light sOCT (vis-sOCT) and other low-coherent techniques have extensively proven their ability for haemoglobin oxygen saturation measurements^7–12^ (stO_2_ = [HbO_2_]/[tHb]). As the stO_2_ is based on the relative ratio between [HbO_2_] and [tHb], stO_2_ measurements primarily require adequate recovery of the shape of the absorption spectrum. On the other hand, the accurate quantification of [tHb] requires adequate recovery of the amplitude of the absorption spectrum. The latter is challenging in sOCT, as this requires precise correction for the scattering contribution to the measured attenuation, as well as the correction for system dependent attenuation parameters such as defocus and the sensitivity roll-off with depth due to the finite pixel size of the detecting spectrograph^13^. Moreover, superior system sensitivity in depth is required to adequately quantify the large optical attenuation of whole blood. As a consequence, current efforts for quantitative [tHb] measurements with sOCT have remained restricted to low haemoglobin concentrations in phantoms^14^ ([tHb] < 6 g/dL) and limited validation on rodent whole blood in terms of sample size and reference methods^8^. These results indicate the great potential of vis-sOCT for the quantification of [tHb], but proof of principle across the clinical [tHb] range of 7–23 g/dL in human whole blood^15^ remains to be given.

Recently, we developed a visible-light sOCT system with superior sensitivity by employing focus tracking and zero-delay acquisition^16^ throughout the entire imaging depth, and we demonstrated its efficacy for the quantification of bilirubin concentrations^13^. In this study, we employ this sOCT system for quantitative [tHb] measurements on human whole blood and compare the results with conventional sOCT, i.e. without focus tracking and zero-delay acquisition. An analysis model is developed to adequately correct for the high scattering contribution by red blood cells to the sOCT signal. We validate our method on human whole blood samples with [tHb] concentrations ranging between 7–23 g/dL.

## Results

### Attenuation coefficient spectra

**Figure 1:**
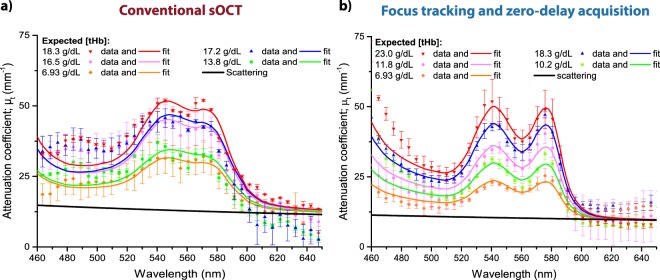
Example of measured and fitted attenuation spectra *μ*_*t*_(λ) for samples with varying [tHb] (average spectrum per sample, error bars represent SD). The plotted scattering spectrum represents the average scattering contribution of all whole blood samples that were included in this study. (**a**) Attenuation spectra obtained by conventional sOCT. (**b**) Attenuation spectra obtained by combining focus tracking and zero-delay acquisition.

Human whole blood samples with varying [tHb] were studied with our visible-light sOCT system to obtain the attenuation coefficient *μ*_*t*_ as a function of wavelength *λ*. This *μ*_*t*_(*λ*) is the sum of the absorption coefficient *μ*_*a*_(*λ*) and the scattering contribution to the signal, which equals the scattering coefficient *μ*_*s*_(*λ*) in the case of single scattering. As an example, typical *μ*_*t*_(*λ*) spectra obtained by both analysis methods are shown in Fig. 1, along with the estimated contribution of scattering. For *λ* < 600 nm, the measured *μ*_*t*_(*λ*) typically increases with expected [tHb]. The attenuation model (Eq. 5) fits better to the measured *μ*_*t*_(*λ*) spectra that were acquired with focus tracking and zero-delay acquisition, with an average (±standard deviation SD) coefficient of determination (R^2^) of 0.91 ± 0.08, versus 0.71 ± 0.17 for the conventional sOCT method. For *λ* > 600 nm, scattering dominates absorption, resulting in a similar *μ*_*t*_(*λ*) across all samples for the focus tracking & zero-delay acquisition method.

### Estimated haemoglobin concentrations

#### Conventional sOCT

**Figure 2:**
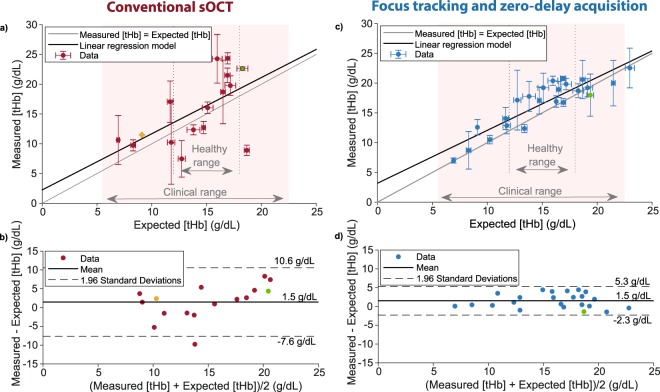
(**a**) Measured versus expected [tHb] values for all samples (average ± SD) by using conventional sOCT (n = 16). (**b**) A Bland-Altman plot for the conventional method shows a bias of 1.5 g/dL and a precision of 9.1 g/dL (1.96 SDs). (**c**) Measured versus expected [tHb] values for all samples (average ± SD) by combining focus tracking and zero-delay acquisition (n = 23). (**d**) The Bland-Altman plot of the data shows a bias of 1.5 g/dL and a precision of 3.8 g/dL. An average of 3 measurements was taken and error bars represent SD. The data points in green were only measured in duplo, while the data point in orange represents a single measurement. As detailed in the methods section, not all samples could be included in the conventional sOCT analysis, due to an excessive loss of signal for the higher [tHb] values.

The measured [tHb] as a function of expected [tHb] is shown in Fig. 2a. The measured and expected values are moderately correlated (Pearson correlation coefficient = 0.59, p = 0.16). Linear regression on the data results in y = 0.94x + 2.25. Figure 2b shows the difference between the measured and expected values as a function of the average of the measured and expected value (Bland-Altman plot), from which we obtain a bias of 1.5 g/dL and a precision of 9.1 g/dL (1.96 SDs).

#### Focus tracking and zero-delay acquisition

Figure 2c shows the measured [tHb] (Eq. 5) as a function of expected [tHb]. The measured and expected values correlate well (Pearson correlation coefficient = 0.89, p < 0.01). Linear regression on the data results in y = 0.89x + 3.18. From the Bland-Altman plot (Fig. 2b) we obtain a bias of 1.5 g/dL (mean) and a precision of 3.8 g/dL.

## Discussion

In this study, we used sOCT to quantify the [tHb] in human whole blood throughout the clinical range. We compared our sOCT method with optimized sensitivity to conventional sOCT, and developed a model to adequately correct for the high scattering contribution of RBCs to the sOCT signal.

In conventional sOCT, the backscattered intensity as a function of depth is not only affected by the sample, but also by the defocus and sensitivity roll-off of the system. Although a correction for roll-off and defocus can be applied to retrieve the sample’s attenuation coefficient, this does not omit the decrease in signal to noise ratio with depth. Our sOCT system overcomes this problem by combining focus tracking with zero-delay acquisition. Compared to conventional sOCT, our method yielded an improved correlation between the attenuation spectra (Fig. 1) and the attenuation model (Eq. 5), and demonstrated improved precision for the [tHb] estimations (Fig. 2). Whereas the conventional method failed to retrieve usable attenuation spectra for those samples with the highest absorption ([tHb] > 19 g/dL), the improved sensitivity of the focus tracking and zero-delay acquisition method resulted in usable data points in this region. We would like to note that the presented comparison is system dependent, as it will be different for sOCT systems with other roll-off and defocus characteristics.

In our comparison to conventional sOCT, we followed a general approach to correct for the OCT system-dependent attenuation in depth^17^. Another method described in literature for *in vivo* whole blood sOCT measurements is division of the backscattered signal inside a blood vessel by the backscattered signal from a certain volume adjacent to that vessel^8^. Whereas this method may be sensitive to tissue inhomogeneities, the *ex vivo* nature of our data did not allow for comparison in this study.

The scattering contribution of blood as determined by our method (~10 mm^−1^) is many times lower than literature values (70–120 mm^−1^)^18^. This can be ascribed to the highly forward directed scattering of RBCs, which causes a large contribution of multiple scattered photons to the OCT-signal. This contribution violates the assumption of single scattering in our Lambert-Beer model (Eq. 1), thereby effectively lowering the measured attenuation^17^. As a consequence, the measured scattering contribution in our measurements is lower than the actual scattering coefficient of whole blood. As the measured scattering contribution by OCT tends to become less dependent on particle concentration for high concentrations^17^, this multiple scattering effect further strengthens our assumption of a constant scattering contribution for all blood samples^18^. As long as the estimation of this scattering contribution to the measured attenuation spectrum is adequate, the estimated absorption coefficient – and thus [tHb] – remain unaffected by multiple scattering: absorption takes place along the photon’s controlled path, which is identical for both single and multiple scattered photons within the coherence length of the sOCT system^19^.

The complexity of flowing human whole blood may influence its optical properties and therefore the precision our [tHb] estimations^18^. For this study, the precision of the [tHb] determinations by sOCT is lower than currently available invasive [tHb] measurement methods and photoplethysmography based non-invasive methods (~2 g/dL based on 1.96 SDs)^4^. There are several reasons for this impaired precision, which may provide room for improvement in future work. Due to the non-Newtonian behaviour of blood, blood flow can induce a non-homogeneous distribution of red blood cells in the radial direction of the capillary. The low shear rate near the wall gives rise to a cell free layer, while an accumulation of cells occurs in the central zone of the capillary^20^. Also aggregates of red blood cells (e.g. rouleaux) can be formed in this central zone of the capillary, and sedimentation due to gravity may occur. All of these effects contribute to the non-homogeneous distribution of blood absorption across the capillary cross section^20^, which will directly affect the measured [tHb]. In this study, we accounted for the cell free layer by excluding the first 15 μm relative to the capillary-sample interface from the measurements. To minimise the effect of the non-uniform distribution of haemoglobin through the capillary, the attenuation coefficient is obtained by fitting Beer’s law (Eq. 1) over a confined depth range of 15–55 μm relative to the capillary-sample interface. As the capillary flow profile can potentially be derived from the OCT-signal directly^21^, this will be an interesting topic for future studies focusing on improved precision of the [tHb] determination. Also other factors of influence, such as vessel depth, vessel diameter and optical density of the tissue covering the vessel, will be important to evaluate.

Although our method of zero-delay acquisition and focus tracking results in optimal sensitivity as a function of measurement depth, it comes at the cost of measurement time. Acquisition of the backscattered spectrum at zero-delay at every depth in the sample requires acquisition of sufficient (~50) lines in order to sample the movement of the piezo driven reference mirror at every depth position. As a consequence, the measurement time scales linearly with the depth interval over which *µ*_*t*_ is measured. In this work, 20 depth positions were used, resulting in a measurement time of 100 seconds per sample. Future *in-vivo* applications require a reduction of measurement time, which can be achieved through optimization of the scanning velocity of the reference mirror, the camera’s line rate (limited by the exposure time), the choice of the depth range and the number of averages, potentially reducing the measurement time by a factor 20. While the first option can be introduced without reducing the signal-to-noise ratio (SNR), the other options will likely reduce the SNR of the system, which may in turn reduce the precision of the [tHb] estimations. Further research is necessary to find an optimal location on the body for future *in-vivo* measurements. Taking the cell free layer into account, blood vessels with a diameter of ≥70 μm will be ideal for *in-vivo* [tHb] determinations. Our method will presumably perform best at a body location where these vessels run as superficial as possible to the skin surface, with low susceptibility for movement artefacts.

## Conclusion

In conclusion, this *ex-vivo* study demonstrates proof of principle for [tHb] measurements by sOCT in human whole blood throughout the clinical range of 7–23 g/dL. With a correlation coefficient of 0.89 between the measured and expected [tHb], sOCT has potential to become a non-invasive alternative for invasive [tHb] determinations, for which future *in-vivo* validation studies will be necessary.

## Methods

### Experimental setup

**Figure 3:**
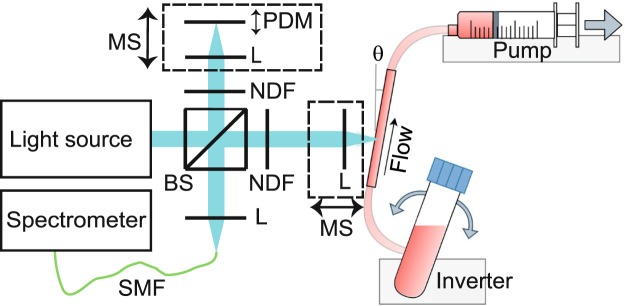
Schematic overview of a blood sample flowing through the sample arm of the sOCT setup. A blood containing tube is placed on an inverter, and blood flows through a glass capillary in which the measurement takes place. A continuous flow is established by a syringe pump. The light is focused inside the capillary by a lens placed on a motorised stage that allows for focus tracking inside the sample. BS: beam splitter, NDF: neutral density filter, L: lens, PDM: piezo driven mirror, MS: motorised stage, SMF: single mode fibre, θ: 10 degree angle.

The sOCT system and data processing have been introduced in detail in Veenstra, *et al*.^13^ and Bosschaart, *et al*.^16^, and will be briefly described next. Compared to our previous work^13^, the sOCT system alignment was modified to increase sensitivity for the longer wavelengths (λ > 600 nm) within the visible wavelength range at the cost of sensitivity for shorter wavelengths (λ < 460 nm). The sOCT system (Fig. 3) had the possibility to acquire the OCT signal as a function of depth with focus tracking and zero-delay acquisition^16^. This optimises system sensitivity and to allows for measurements of the sample’s optical attenuation coefficient *µ*_*t*_ independent of system parameters^13^. The light of a supercontinuum light source (SuperK EXTREME EXB-6, NKT Photonics, Denmark) was short pass filtered at 700 nm (FESH0700, Thorlabs, USA) after which a beam splitter (BS028, Thorlabs, USA) guided 90% and 10% of the light towards the reference and sample arm, respectively. Achromatic lenses (AC127-025-A, Thorlabs, USA) focused the light on the sample contained by a glass capillary in the sample arm (Fisher Scientific B.V., The Netherlands, inner diameter 1.2 mm, outer diameter 1.8 mm), and a piezo-driven oscillating mirror in the reference arm. The Rayleigh length of the foci was 55 µm at λ = 550 nm. The optical power incident on the sample was ~6 mW. A motorised linear stage (T-LS13M, Zaber, USA) controlled the reference arm length by joint translation of the reference mirror and its focusing lens. An identical stage facilitated focus tracking by translation of the focusing lens in the sample arm. The back scattered light from both arms was guided by a single mode fibre (S405-XP, Thorlabs, USA) to a home-built spectrometer, where a grating dispersed the light onto a line scan camera (Sprint spL4096-140 km, Basler, Germany) resulting in spectral resolution δλ = 0.1 nm. The detected spectrum ranged from 460 to 650 nm, resulting in a coherence length of 1.4 µm in air. The sensitivity of the system was estimated at 104.7 dB, with an −3 dB sensitivity roll-off depth of 307 µm when zero-delay acquisition was not applied.

### Focus tracking and zero-delay acquisition

Due to the finite pixel size of the detector, sOCT comes with a strong sensitivity roll-off as a function of optical path length difference (∆OPL) between both arms, especially in the visible wavelength region. To acquire the OCT-signal with maximum spectrograph sensitivity across all depths, we performed acquisition at ΔOPL = 0 (zero-delay) at any depth inside the sample. As a consequence, the unwanted crosstalk between the OCT-signal, its complex conjugate and the DC-component around zero-delay needed to be removed. We followed the same approach as in our previous work^13,16^, where the piezo-driven oscillating reference mirror modulated the optical path length difference between the sample and reference arm, resulting in a Doppler shift with frequency *f*_*d*_ = 2 *v*_*r*_/*λ*. Here *v*_*r*_ = 0.85 mm/s was the velocity of the reference mirror and the spatial scanning range of the mirror was 5 μm^13^. By applying a rectangular bandpass filter around the negatively shifted frequencies in the Fourier-domain (−7.3 to −0.4 kHz), the DC-component and the complex conjugate could be effectively removed from the OCT-signal^13^.

#### Short time Fourier transformation

Prior to each measurement, zero-delay and focus position were matched at a depth of 15 µm relative to the sample-capillary interface. Spectroscopic detection of a total of *N* = 2500 lines (exposure time: 50 µs per line, line rate: 16.7 kHz) was performed. Next, short time Fourier transformation resulted in a spatially and spectrally confined dataset (spectral resolution 5 nm, spatial resolution ranging between 15 μm at 460 nm and 30 μm at 650 nm) from which the backscattered spectrum at zero-delay *S(λ*) could be isolated using the procedure described above. Translation of both zero-delay and focus position by the motorised stages allowed for acquisition of *S(λ*) at any depth. This procedure was repeated until the complete desired depth range was covered, resulting in a spectrally and spatially resolved dataset of backscattered light from the sample *S*(*λ, d)*.

#### Retrieving the attenuation spectrum

Prior to attenuation analysis, *S*_*conv*_(*λ, d)* was corrected for the OCT-system dependent attenuation similar to the approach in Almasian, *et al*.^17^. A wavelength dependent correction for the sensitivity roll-off of the system was applied by dividing *S*_*conv*_(*λ, d*) by the relative sensitivity of the system as a function of depth^22^ for every wavelength. Under the assumption of single scattering, the attenuation coefficient *μ*_*t*_*(λ*) of the sample was obtained by fitting a linear Lambert-Beer model to the natural logarithm of *S*(*λ, d*)^13^:

1 $$\mathrm{ln}({(S(\lambda ,d)-{S}_{bg}(\lambda ))}^{2})=\,\mathrm{ln}(\alpha (\lambda ))-2{{\mu }}_{{t}}(\lambda )d$$

with *α*(*λ*) and *μ*_*t*_*(λ*) free running fit parameters and *S*_*bg*_(*λ*) a background term that was obtained by measuring *S*(*λ)* at a depth of 1000 µm inside the blood sample, where light had been fully attenuated.

### Conventional sOCT

To investigate the advantages of our focus tracking and zero-delay acquisition method, we used a conventional sOCT method without reference mirror oscillation and focus tracking for comparison. To prevent the complex conjugate from interfering with the signal, the surface of the sample and the focus position were matched at a depth of 100 µm relative to zero-delay. Similar to the method described above, spectroscopic detection of a total of *N* = 2500 lines (exposure time: 50 µs per line, line rate: 16.7 kHz) was performed and the spectra were corrected for the background by subtracting the signal measured at a depth of 1000 µm inside the sample. To ensure the most robust fitting procedure for the Lambert-Beer model (below) with the most stable outcome for *μ*_*t*_(*λ)*, short time Fourier transformation was performed with a larger spectral window (20 nm) compared to our focus tracking and zero-delay method (5 nm). This resulted in a spatially and spectrally resolved dataset *S*_*conv*_(*λ, d*) with a spatial resolution that ranged from 3.8 µm at 460 nm to 7.5 µm at 650 nm. A 75% overlap for the spectral window yielded an effective 5 nm spectral spacing between the data, identical to the focus tracking and zero-delay acquisition method.

#### Retrieving the attenuation spectrum

Correction for the sensitivity roll-off of the system was applied by dividing *S*_*conv*_(*λ, d*) by the relative sensitivity of the system as a function of depth^22^ for every wavelength. Under the assumption of single scattering, the attenuation coefficient *μ*_*t*_(*λ*) was obtained similar to Eq. 1 by fitting a Lambert-Beer model to the background corrected *S*_*conv*_(*λ, d*):

2 $$\mathrm{ln}({S}_{conv}{(\lambda ,d)}^{2})=\,\mathrm{ln}(\alpha (\lambda ))-2{{\mu }}_{{t}}(\lambda )d$$

with *α*(*λ*) and *μ*_*t*_(*λ*) free running fit parameters.

### Estimating chromophore concentrations

For both sOCT methods, we modeled the obtained total attenuation coefficient as the sum of a scatter power function *aλ*^*−b*^ and the individual absorption spectra of HbO_2_ and Hb in whole blood:

3 $${{\mu }}_{{t}}(\lambda )=a{\lambda }^{-b}+\sum _{i}{C}_{i}{{\mu }}_{a,i}(\lambda )$$

with scaling factor *a*, scatter power *b* and *C*_*i*_ the concentration of the i-th chromophore relative to the reference absorption spectrum *μ*_*a,i*_(*λ*).

In blood, the major fraction of haemoglobin is located inside RBCs rather than being homogeneously distributed throughout the sample. The fraction of light that does not encounter RBCs will not be affected by absorption, causing the absorption to scale non-linearly with concentration. This phenomenon is known as absorption flattening, and can be described by^18,23^:

4 $${{\mu }}_{a,effective}=(\frac{1-\exp (\,-\,{{\mu }}_{a,free}{d}_{rbc})}{{{\mu }}_{a,free}{d}_{rbc}}){{\mu }}_{a,free}$$

where *μ*_*a,free*_, is the absorption coefficient of an absorber when freely present in a solution and *μ*_*a,effective*_ the effective absorption coefficient if the absorber is located inside particles. In the derivation of Eq. 4 RBCs were modelled as cubes with edge length ${d}_{rbc}=\sqrt[3]{90}$ μm^18^. Combining Eqs 3 and 4 results in:

5 $${{\mu }}_{t}(\lambda )=a{\lambda }^{-b}+\sum _{i}(\frac{1-\,\exp (-{C}_{i}{{\mu }}_{a,i}(\lambda ){d}_{rbc})}{{d}_{rbc}})$$

which is the model for the total attenuation as used throughout this work. The non-flattened reference spectra *μ*_*a,i*_ used in this work were calculated by solving Eq. 4 for *μ*_*a,free*_ after inserting the (flattened) HbO_2_ and Hb absorption spectra in whole blood from Bosschaart *et al*.^18^ as *μ*_*a,effective*_.

Due to the high amount of dependent scattering by RBCs, scattering can be assumed to be constant within the haematocrit range associated with the measured haemoglobin concentrations (hct ≈ 21–69%)^18^. We therefore used a single set of scaling factor *a* and scatter power *b* to correct all measured *μ*_*t*_*(λ)* for scattering. The values for *a* and *b* were obtained by non-linear least squares fitting of Eq. 5 to the average *μ*_*t*_*(λ)* of all measurements in the 460–650 nm wavelength range. The lower and upper limits of all fit parameters (*a*, *b* and *C*_*i*_) were set to 0 and infinity, respectively. This procedure resulted in an estimated contribution of scattering to the total attenuation modelled by *μ*_*s*_ [mm^−1^] = 258.2*λ*^−0.5165^, with *λ* in nm. This leads to a *μ*_*s*_ ranging from 9.1 mm^−1^ to 10.9 mm^−1^ for the 460–650 nm wavelength range.

Chromophore concentrations were obtained by fitting Eq. 5 to *μ*_*t*_*(λ)* in the wavelength range 520–590 nm for each measurement separately, with *a* and *b* as determined by the procedure in the previous paragraph and *C*_*i*_ as the only fit parameter with limits 0 – infinity. The [tHb] was obtained by summing the individual contributions of [HbO_2_] and [Hb].

### Human whole blood samples

A 15 mL falcon tube filled with 9 mL blood sample was placed on an inverter (frequency 0.1 Hz), to prevent sedimentation of RBCs and to ensure continuous mixing of the blood throughout each measurement. A syringe pump (AL1000-220, World Precision Instruments, USA) established a blood flow speed of 0.3 mL/min through the glass capillary, which was placed inside the sample arm. The capillary was placed under an angle of ~10 degrees relative to the incident beam to prevent detection of specular reflections from the glass capillary. Measurements were performed in a depth range of 15–55 μm (step size 2 μm) relative to the capillary-sample interface.

To validate our focus tracking and zero-delay acquisition method, we created and measured a series of 23 blood samples with varying expected [tHb] in the range of 6.9–23.0 g/dL. For 18 of these blood samples ([tHb] from 6.9 to 21.5 g/dL), the OCT signal was also acquired without reference mirror oscillation, allowing for a comparative analysis with the conventional sOCT method. All human whole blood samples were prepared, using the blood of six anonymized, healthy donors from the Experimental Centre for Technical Medicine (ECTM) of the University of Twente. All methods were carried out in accordance with relevant guidelines and regulations. In agreement with the Declaration of Helsinki, informed consent was obtained from all volunteers and the used blood collection procedure was approved by the local Medical Research Ethics Committee (METC Twente). The [tHb] of the samples was varied by diluting the blood with PBS, as well as thickening the blood by removing plasma. Visual inspection of the plasma showed no signs of haemolysis in all samples. All blood samples were exposed to air during preparation and contained Heparin as anticoagulant. Expected [tHb] values for all samples were obtained by reference measurements using a blood analyser (Avoximeter 1000E, Instrumentation Laboratory, USA). Both the sOCT and reference measurements were performed in triplo. One focus tracking and zero-delay acquisition and one conventional sOCT measurement (expected [tHb] = 19.4 g/dL) were excluded from this study after visual inspection of the A-scans, which revealed that no scattering medium (i.e. blood) was present inside the measurement volume – most likely to be caused by an obstruction inside the capillary. As a result, these data points were measured only in duplo. For the conventional sOCT method, two samples with high haemoglobin concentrations were excluded, as no useable attenuation spectra could be obtained due to excessive signal loss (expected [tHb] of 19.1 g/dL and 21.5 g/dL). For one other sample in the conventional sOCT analysis, two out of three measurements (expected [tHb] = 9.10 g/dL) had to be excluded due to an unforeseen interference of the complex conjugate with the signal, leaving a single measurement for this data point.

## Data Availability

The datasets generated during and/or analysed during the current study are available from the corresponding author on reasonable request.
